# Effects of vitamin D supplementation on advanced glycation end products signaling pathway in T2DM patients: a randomized, placebo-controlled, double blind clinical trial

**DOI:** 10.1186/s13098-019-0479-x

**Published:** 2019-10-26

**Authors:** Mahsa Omidian, Mahmoud Djalali, Mohammad Hassan Javanbakht, Mohammad Reza Eshraghian, Maryam Abshirini, Parisa Omidian, Ehsan Alvandi, Maryam Mahmoudi

**Affiliations:** 1 Department of Cellular and Molecular Nutrition, School of Nutritional Sciences and Dietetics, Tehran University of Medical Sciences, Poorsina Street, Enghelab Avenue, PO Box: 14155-6446, Tehran, Iran; 2 Department of Biostatistics, School of Public Health, Tehran University of Medical Sciences, Tehran, Iran; 3 Department of Community Nutrition, School of Nutritional Sciences and Dietetics, Tehran University of Medical Sciences, Tehran, Iran; 4 Rasoul Akram Complex Hospital, Iran University of Medical Sciences, Tehran, Iran

**Keywords:** Vitamin D, Diabetic complications, GLO1, AGEs, RAGE, TNF-α

## Abstract

**Background:**

Several researches have recommended vitamin D possible health benefits on diabetic complications development, but a few number of studies have been accomplished on the molecular and cellular mechanisms. Certain cellular pathways modification and also some transcription factors activation may protect cells from hyperglycemia condition induced damages. This study purpose was to determine the vitamin D supplementation effect on some key factors [advanced glycation end products (AGEs) signaling pathway] that were involved in the diabetic complications occurrence and progression for type-2 diabetes participants.

**Methodology:**

48 type-2 diabetic patients (T2DM) randomly divided into two groups (n = 24 per group), receiving: 100-µg vitamin D or placebo for 3 months. At this study beginning and the end, the receptor expression for advanced glycation end products (RAGE) and glyoxalase I (GLO1) enzyme from peripheral blood mononuclear cells (PBMCs) and AGEs and tumor necrosis factor-α (TNF-α) serum levels were measured by the use of real-time PCR and ELISA methods, respectively.

**Results:**

This study results demonstrated that vitamin D supplementation could down-regulate RAGE mRNA [fold change = 0.72 in vitamin D vs. 0.95 in placebo) P = 0.001)]. In addition, no significant changes were observed for GLO1 enzyme expression (P = 0.06). This study results also indicated that vitamin D serum level significantly increased in vitamin D group (P < 0.001). Moreover, AGES and TNF-α serum levels significantly reduced in vitamin D group, but they were remained unchanged in the placebo group.

**Conclusion:**

In conclusion, vascular complications are more frequent in diabetic patients, and vitamin D treatment may prevent or delay the complications onset in these patients by AGEs serum level and RAGE gene expression reducing.

*Trial registration* NCT03008057. Registered December 2016

## Background

Type 2 diabetes mellitus (T2DM) is now epidemic in all over the world. The diabetes mellitus global prevalence amongst adult population (with the age from 20 to 79 years old) was reported as 425 million in 2017, and it is estimated to reach 629 million by 2045. Despite of the different type of drugs developing in order to control diabetes, diabetes complications impose huge costs on governments [1]. Chronic hyperglycemia plays a significant role in these complications occurrence and progression. Hyperglycemia appears to cause epigenetic changes, which affect the gene expression in an extensive range of cells. It has been postulated that advanced glycation end products (AGEs) and it’s certain receptor (RAGE) pathway is one of the major pathways involved in diabetic complications [2].

The AGEs formation rate is accelerated by hyperglycemia. AGEs are formed as a result of non-enzymatic and covalent reactions between sugars reducing and amine groups on proteins, nucleic acids, or lipids [3].

Following this process, the extracellular matrix structure and function are affected, consequently. For example, cross-linking between AGEs and type I collagen and elastin results in declined blood vessels compliance and also enhanced vascular stiffness [4]. In addition, AGEs activate cellular signaling pathways in order to be linked with enhanced production of free radicals and inflammatory processes, through interacting with RAGE. The RAGE signaling pathway results in inducing nuclear factor-κβ (NF-κB) translocation that increased transcription of endothelial dysfunction biomarkers including intercellular adhesion molecule-1, endothelin-1, and E-selectin [5, 6].

Due to induction of endothelial dysfunction biomarkers, diabetic patients are more prone to atherosclerosis in comparison with those non-diabetic subjects. In addition to its function as an endothelial dysfunction biomarkers activator, AGEs impair endothelial nitric oxide synthase activation, and also contribute to plaque destabilization [7].

Animal studies have indicated that AGEs induce glomerulosclerosis, and tubulointerstitial fibrosis, which consequently, result in nephropathy in diabetic patients [8, 9]. Retinal blood vessels are also considered as target for AGE-mediated damages [10]. Therefore, it is necessary to develop therapeutic strategies for AGEs degradation and prevention of diabetic complications due to AGEs–RAGE interaction harmful effects.

Previous evidence has demonstrated that vitamin D supplementation could reduce some diabetes complications like retinopathy and nephropathy, but various mechanisms that are involved in this effect have not been completely clarified, yet [11, 12]. The glyoxalase I enzyme (GLO1) is a recognized as the glyoxalase system main enzyme (glyoxalase I, glyoxalase II and reduced glutathione), which is involved in methylglyoxal and AGEs degradation and elimination from body. Human glyoxalase I is a dimeric Zn (2+) metalloenzyme, and was expressed in different cell types like immune cells and endothelial cells. Glyoxalase-1 increased expression reduces the inflammatory and oxidative processes that were caused by AGEs [13, 14]. It has also been indicated that TNF-α is a RAGE gene expression regulator [15]. Vitamin D has already the ability to decrease serum TNF-α levels [16]. We hypothesize that vitamin D may regulate those downstream factors protecting cells against damages. This study purpose was to assess the vitamin D supplementation effectiveness on GLO1 and RAGE genes expression and also on AGES and TNF-α serum level in type 2 diabetic patients.

## Methodology

### Participants and study design

Forty-eight patients with type 2 diabetes attending Iranian Diabetes association (IDA) in Tehran were enrolled in this double blind placebo-controlled clinical trial.

Diabetes type 2 was diagnosed by an endocrinologist, with respect to the American Diabetes Association criteria with higher than 126 mg/dl fasting blood sugar (FBS) concentrations (confirmed by twice testing without using antiglycemic medications), and at least by passing 2 years from the disease diagnosis time.

Inclusion criteria were: aged 30–60 years old, consuming a stabilized dose of oral anti-diabetic drugs and statins history, willing for participating, having 20 to 30 kg/m^2^ body mass index (BMI), receiving no herbal products or dietary supplements at least 3 months before and also in the meantime of the trial, willing to maintain their current diet, physical activity and also their life style during this study 12 weeks period. These patients excluded from this study: patients consuming vitamin D supplements within 3 months, having diabetes complication, Using insulin or thiazolidinediones or anti-obesity drugs, pregnancy or lactation, having a clinical disease history like malabsorption, type 1 diabetes, pancreatitis, liver damage, inflammatory diseases, and malignancy, consuming some drugs that could interact with vitamin D absorption including anti-consultants drugs (phenytoin and phenobarbital) and also those patients who smoked. The exclusion criteria were any changes in the medications type or dosage during this study, and lack of adherence to the trial in terms of refusing to consume at least 90% of recommended treatments.

This study was approved by the local Ethical Committee of Tehran University of Medical Sciences (reference number: 32615). Moreover, this trial was registered at ClinicalTrials.gov (identifier: NCT03008057). The specialists and staffs were blinded during this trial.

### Randomization and intervention

Forty-eight patients were included in this study. We utilized stratified randomization in terms of sex (male/female) and BMI (normal/overweight). This study participants were randomly divided into two groups (vitamin D and placebo) by permuted blocks using computer-generated random sequences. In this study, the vitamin D and placebo supplementation groups’ ratio was 1:1. An assistant performed the block randomization, and the intervention was blinded for both researchers and patients. The participants were assigned into two groups receiving vitamin D supplements or placebo by random. Vitamin D and placebo tablets were purchased from the Pars Minoo Pharmaceutical, Cosmetic and Hygienic Company (Iran). Each one of vitamin D tablets contains 100 μg or 4000 IU of vitamin D. Each placebo tablet contains gelatin starch, lactose powder, magnesium stearate, and citric acid. The lactose powder percentage has reduction in vitamin D supplements, and vitamin D was added instead of it. Placebo and vitamin D tablets have the same shape, size, and the color. The type of supplements was blinded as A and B packages for researchers and patients. Simultaneously, both groups’ participants were advised to continue their usual anti-diabetic drugs, usual diets and physical activity.

### Sample blood collection and peripheral blood mononuclear cells (PBMCs) separation

At this study beginning and the end, after 12 to 14 h fasting 15 ml of venous blood was taken from patients. 10 ml of blood samples were transferred to sterile tubes containing EDTAK3 anticoagulant for isolating peripheral blood mononuclear cells (PBMCs), and 5 ml was transferred into gelatinous tubes for serum separation. Serum samples were separated by centrifugation at 3000 RPM for 15 min and then were stored at − 80 °C, for accomplishing further assays.

Immediately after taking blood samples, PBMCs were isolated from EDTAK3 peripheral blood using the Ficoll (Lymphodex, Inno-Train, Germany) and standard density gradient centrifugation.

### RNA extraction and cDNA synthesis

RNA was extracted and purified by the use of Hybrid-R Blood RNA Kit (GeneAll Biotech, South Korea) with respect to kit’s protocol. In the next step, the RNAs quantity and purity were evaluated using a NanoDrop spectrophotometer (NanoDrop™ 2000/2000c Technologies, Wilmington, DE, USA) and a ratio of 260/280 nm ranged from 1.8 to 2.2 was considered as pure extracted RNAs. The isolated RNA was treated with DNAse. We designed primers specifically for RNA and performed using exon overlapping primer design even after DNAse treatment. We used 500 ng mRNA for retro transcription with respect to cDNA kit protocol.

cDNA synthesis kit (TaKaRa Bio Inc., Japan) was utilized in order to accomplish single strand complementary DNA (cDNA) synthesis. After that, cDNA were stored at − 20 °C, for performing further assays by the use of real time-PCR method.

### Real-time polymerase chain reaction

The RAGE, GLO1, and β-actin genes primers were designed using Allele ID 6 software (PREMIER Biosoft International 3786 Corina Way Palo Alto CA 94303-4504, USA). The primer blast software was applied in order to check the primer accuracy that were bind to the target position, and not to link it to other locations. 22 placebo group samples and 23 vitamin D group samples were examined in the mRNA expression analysis using qPCR, and also three replicates per sample have been tested along with that.

In addition, β-actin gene was utilized as housekeeping. The primers sequence is presented in Table 1. For gene expression, real-time PCR was performed using the Step One system (Applied Biosystems, Foster City, CA, USA), real-time PCR system and SYBR Green detection method [17]. In the following, all gene expression data were normalized to β-actin (ΔCT). The fold changes were calculated by the use of the 2^−ΔΔCT^ method.

**Table 1:** Primers used for this study

Gene name	Sequence
RAGE	Forward: 5′-**GCAGTCGGAGCTAATGGTG**-3′
	Reverse: 5′-**AGGTCAGGGTTACGGTTCCA**-3′
GLO-1	Forward: 5′-**CAGACCATGCTACGAGTGA**-3′
	Reverse: 5′-**GGTCTCATCATCTTCAGTGC**-3′
β-actin	Forward: 5′-**TGGCACCCAGCACAATGAAG** -3′
	Reverse: 5′-**AGTCATAGTCCGCCTAGAAGC**-3′

### Outcomes and measurements

Primary outcomes were described as AGEs, TNF-α, FBS, and Hba1c levels. Secondary outcomes included gene expression of RAGE and GLO1. The anthropometric parameters were measured before and after the intervention, with respect to standard protocols. Body mass index (BMI) was computed as body weight (kg) divided by the height square (m). FBS levels were measured applying enzymatic method by the use of autoanalyzer instrument. HbA1c percentages were measured in all of the blood samples by immunoturbidimetric method. Serum AGEs and TNF-α were measured by the use of ELISA Kits (Shanghai Crystal Day Biotech Co., Ltd, China). Two measurements per sample have been accomplished in the TNF-α and AGEs determinations by ELISA. Intra and inter-assay coefficients of TNF-α and AGEs variation were < 8% and < 10% using ELISA kits, respectively.

At this study beginning and the end, a set of questionnaires were completed by interviews including general information questionnaire, and short-form International Physical Activity Questionnaire (IPAQ) [18].

### Ethical considerations

1. Provide descriptions of study protocol to patients.
2. Obtaining written consent [approved by the local Ethical Committee of Tehran University of Medical Sciences (reference number: 32615)] from participants.
3. Ensure about that this dose of supplementation with vitamin D is not harmful for patients, with respect to previous studies [19].
4. No change in the patient’s treatment protocols provided by their physician.
5. Patient’s information confidentiality.
6. Enabling patients to withdraw from the study whenever they desired.

### Data analysis

The continuous variables normality was determined using Kolmogorov–Smirnov distribution test. The logarithmic transformations were used in order to analyze those data that had not normal distribution. The groups continuous and categorical baseline characteristics were assessed using a t test and a Chi-square test, respectively. Quantitative data were expressed as mean ± SD. Within-group (before/after intervention) differences were compared by the use of paired sample t-test. Comparison of the end of trial outcomes values of these two groups was made by the ANCOVA test in order to control the covariate possible confounding effect. Mean changes in biochemical variables were compared between the groups by the use of Independent Samples t Test. SPSS version 16 (SPSS Inc., Chicago, IL, USA) was applied for data analyzing. P-value < 0.05 was considered statistically significant in all analysis.

## Results

116 T2DM patients were volunteered to participate, 68 subjects excluded during the screen due to already on vitamin D or insulin, as shown in Fig. 1 from October 2017 to May 2018. Finally, 48 subjects were included who were eligible and willing to participate in the intervention. During the study, two subjects from the vitamin D and placebo groups withdrew from the study for personal reasons and change of medications type, respectively, so 46 subjects were included finally, and completed (23 in vitamin D supplement group and 23 patients in placebo group) the study with respect to the study program.

**Fig. 1:**
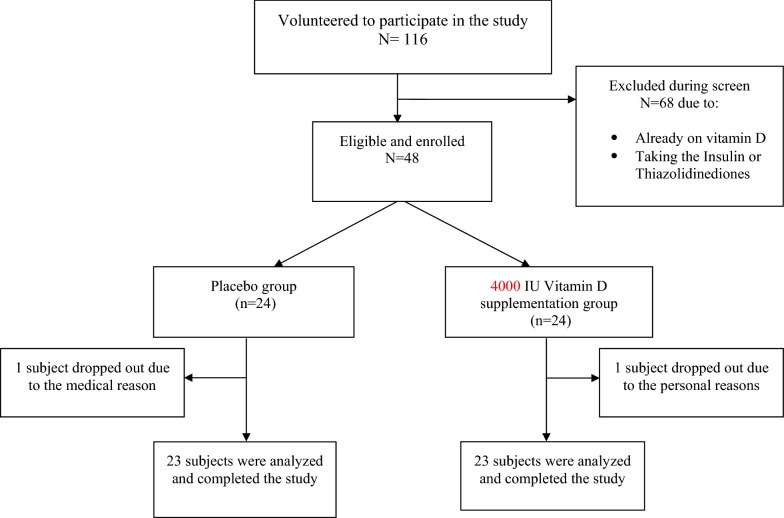
Trial profile

### Baseline

Table 2 displayed baseline characteristics in the intervention and control groups. No significant difference was observed between the characteristics at the baseline. However, HbA1c level and metformin dose was higher in placebo group in comparison with intervention (P = 0.06 and P = 0.01), respectively.

**Table 2:** Baseline characteristics of participants

	Placebo (n = 23)	Vitamin D2 (n = 23)	P-value
Sex, n (%)			0.7
Male	11 (23.9)	12 (26.1)	
Female	12 (26.1)	11 (23.9)	
Age (year)	52.4 ± 5.7	51.3 ± 4.7	0.4
Educational level, n (%)			0.5
Primary	6 (13.0)	4 (8.7)	
Diploma	10 (21.7)	6 (13.0)	
Bachelor or master degree	7 (15.2)	13 (28.8)	
Employed, n (%)			0.7
Yes	14 (30.4)	15 (26.3)	
No	9 (19.7)	8 (17.4)	
Aspirin use, n (%)			0.7
Yes	10 (21.7)	11 (23.9)	
No	13 (28.3)	12 (26.1)	
Diabetes duration (year)	6.7 ± 3.0	5.8 ± 2.6	0.3
WC (cm)	97.2 ± 8.5	96.6 ± 7.4	0.8
BMI (kg/m^2^)	27.5 ± 1.6	26.8 ± 1.4	0.6
Physical activity, n (%)			0.2
Mild	12 (26.1)	17 (37)	
Moderate	10 (21.7)	6 (13)	
High	1 (2.2)	0 (0)	
HbA1c (%)	7.9 ± 1.1	7.4 ± 0.8	0.06
Metformin dose (mg/day)	1282.6 ± 253.4	1065.2 ± 274	0.01
Gliclazide dose (mg/day)	73.1 ± 29.1	70 ± 36.8	0.7

Quantitative variables are presented as mean ± SD, and categorical variables are expressed by frequencies (%)

Student t-test was applied for variable with normal distribution

Chi-square and Fisher exact tests were performed for categorical variables

*WC* waist circumference, *BMI* body mass index, *HbA1c* hemoglobin A1c

### Post-intervention

The mean of BMI, WC, and the FBS, TNF-α, AGEs and 25(OH) D serum level are indicated in Table 3. The results demonstrated that serum levels of TNF-α and FBS, and AGEs significantly decreased (P < 0.004) in vitamin D group after receiving supplements, while 25(OH) D serum level significantly increased after receiving supplement in intervention group (P < 0.001). However, there were no significant differences in serum levels of FBS (P = 0.4), TNF-α (P = 0.1), AGEs (P = 0.7), and 25(OH) D (P = 0.08), by passing 3 months in placebo group. The FBS, TNF-α, AGEs serum level in intervention group was significantly lower in comparison with placebo (P < 0.03) after 3 months supplementation. There were observed no significant difference in the BMI and WC means at the baseline and also by passing 3 months from supplementation in both placebo and intervention groups. Three months of supplementation did not cause significant change in BMI and WC mean.

**Table 3:** Comparison of the initial and final values of the variables under study in the two groups

Characteristics	Vitamin D group n = (23)	Placebo group N = (23)	*P* value
BMI (kg/m^2^)			
Before	26.8 ± 1.4	27.5 ± 1.6	0.6
After	26.6 ± 1.3	27.3 ± 1.7	0.3
Difference	− 0.06 ± 0.12	− 0.06 ± 0.10	0.9
*P* value	0.6	0.4	
WC (cm)			
Before	96.6 ± 7.4	97.2 ± 8.5	0.8
After	96.5 ± 7.5	97.1 ± 8.4	*0.8*
Difference	− 0.13 ± 0.15	− 0.20 ± 0.21	0.8
*P* value	0.4	0.3	
FBS (mg/dl)			
Before	169.5 ± 35.5	186.5 ± 52.8	0.2
After	148.6 ± 36.3	179.6 ± 49.7	0.02
Difference	− 20.8 ± 6.3	− 6.8 ± 9.5	0.03
*P* value	0.003	0.4	
TNF-α (ng/l)			
Before	247.1 ± 71.4	279.3 ± 97.0	0.2
After	188.8 ± 60.9	250.7 ± 78.1	0.004
Difference	− 58.2 ± 10.7	− 28.7 ± 17.5	0.007
*P* value	< 0.001	0.1	
AGE (U/ml)			
Before	612.1 ± 254.7	623.4 ± 238.6	0.9
After	413.7 ± 146.9	597.7 ± 240.0	0.003
Difference	− 199.2 ± 52.2	− 25.7 ± 69.1	0.001
*P* value	0.001	0.7	
Serum 25(OH) D (ng/ml)			
Before	14.7 ± 9.9	14.8 ± 13.1	0.7
After	30.2 ± 9.2	16.8 ± 12.9	< 0.001
Difference	15.4 ± 1.2	1.9 ± 1.08	< 0.001
*P* value	< 0.001	0.374	

Italic value indicates the significance of *P* value < 0.05

All values are expressed as mean ± SD

Pair t test was used to compare the within group (before/after intervention) differences in both group

Independent t test was used to compare the mean values of the parameters between the groups

ANCOVA was applied to compare the mean differences between the two groups to control the confounding effect of metformin and HbA1c level

*BMI* body mass index, *WC* waist circumference, *FBS* fasting blood sugar, *TNF-α* tumor necrosis factor alpha, *AGEs* advanced glycation end products

Because vitamin D group had a significant lower intake of metformin dose and HbA1c level at the baseline, consequently ANCOVA test was applied in order to control this effect on the mean differences. As indicated, the FBS, TNF-α, AGEs adjusted mean differences were significantly higher in intervention group in comparison with placebo group (P < 0.04). In addition, the mean difference of serum levels of 25(OH) D was significantly higher in intervention group (P-value < 0.001) compared to placebo.

### RAGE and GLO-1 gene expression in isolated PBMCs

**Fig. 2:**
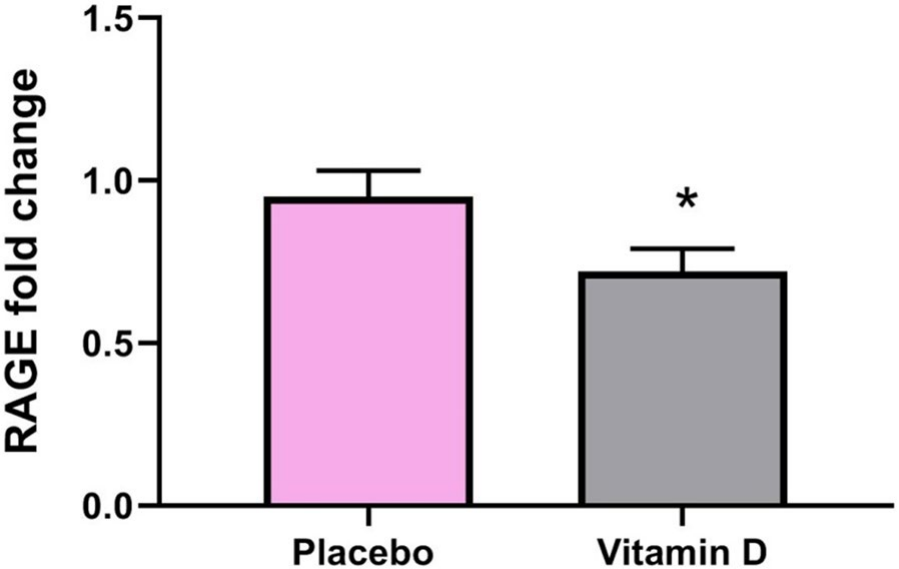
Effect of 12-week supplementation with vitamin D or placebo on expression ratio of the receptor for advanced glycation end products (RAGE) gene in PBMCs of T2DM patients. Error bars indicate standard error of the mean. Independent t-test was used (P-value = 0.001, * < 0.05)

**Fig. 3:**
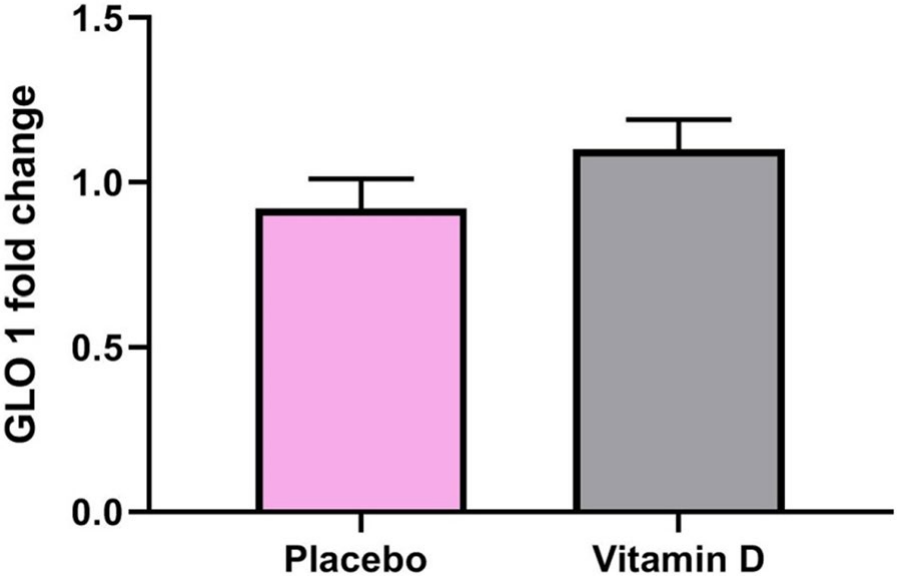
Effect of 12-week supplementation with vitamin D or placebo on expression ratio of glyoxalase I (GLO 1) gene in PBMCs of T2DM patients. Error bars indicate standard error of the mean. Independent t-test was used (P-value = 0.06)

As indicated in Fig. 2 vitamin D decrease RAGE gene expression (fold change 0.72 in vitamin D vs. 0.95 in placebo) in PBMCs of T2DM patients. However, GLO-1 gene expression did not significantly change after interventions (Fig. 3). Our results also showed that change in RAGE gene expression remained significant (P = 0.006) even after adjustment for baseline values of gene expression.

## Discussion

48 patients were enrolled in this study, in a 12 weeks vitamin D, and placebo clinical trial in order to evaluate the vitamin D supplementation effect on AGEs and TNF-α serum levels, and also on RAGE and GLO-1 gene expression. Vitamin D can significantly reduce AGEs and TNF-α serum levels and gene expression of RAGE in PBMCs of T2DM patients with vitamin D deficiency or insufficiency (serum levels of 25(OH) vitamin D_3_ < 30 ng/mL). AGEs play a crucial role in both diabetes micro-vascular and macro-vascular complications [9].

AGEs contribute to develop diabetic angiopathy throughout two important mechanisms: AGEs compounds accumulate in the vessel wall and modify cellular structure. Moreover, AGEs after interaction with RAGE can alter cellular function and cellular proliferation [20]. RAGE receptors are expressed on the various types of tissues and cells surface, and interaction between AGEs and RAGE result in altering vascular homeostatic functions. RAGE–AGE engagement has been thought to play an essential role in diabetic angiopathy [21]. Accumulating data has reported that several AGEs inhibitors like pyridoxamine and LR-90 could prevent the vascular disease from progressing in rats [22, 23].

Many studies assessed the relationship between diabetes and vitamin D [24–26]. Vitamin D receptors exist in various body cells, and can regulate molecular pathways and transcription factors [27]. Vitamin D has already been reported as being able to attenuate AGEs toxic effects in vitro [28]. For this reason, we examined vitamin D effect on AGEs–RAGE system as a therapeutic target for preventing from diabetic complications. This study results demonstrated that vitamin D could significantly reduce RAGE gene expression in PBMCs of diabetic patients. Furthermore, RAGE expression modulation may improve vascular oxidative stress and alleviate cardiovascular complications [29]. A few studies have detected vitamin D supplementation effect on RAGE expression, which indicates conflicting results in various tissues and cells [30, 31]. Also in this study, vitamin D supplementation for duration of 3 months could significantly reduce TNF-α serum levels. There are also several studies with similar results that are consistent with this finding. In meta-analysis conducted by Mousa et al. [32] there was a significant improvement in TNF-α level after vitamin D supplementation in T2DM patients. Several proposed mechanisms for vitamin D beneficial effects on RAGE expression in this study are as followings: Vitamin D can effectively reduce TNF-α and AGEs serum levels. With respect to earlier in vivo evidences, AGEs and tumor necrosis factor-α (TNF-α) can up-regulate the RAGE gene expression. Another recommended mechanism for vitamin D beneficial effect on RAGE expression is associated to the mitochondrial dysfunction and oxidative stress attenuation. Hyperglycemia induce mitochondrial dysfunction and oxidative stress that are associated with RAGE over expression [33]. The third mechanism is associated to vitamin D protective effect on soluble receptor for AGEs (sRAGE). sRAGE is identified as a binder to AGEs, and also as an AGE–RAGE system inhibitor. Past clinical trial has indicated that vitamin D could increase sRAGE serum level in hemodialysis patients [34].

With respect to previous studies, over expression of GLO1 can effectively reduce AGEs formation [35]. In this study, the GLO1 expression level in PBMCs of T2DM indicated no change after receiving vitamin D. Up to now; only one study has examined the vitamin D effect on GLO1 expression with conflicting results in different cells [13]. Therefore, further studies are required in this field.

In conclusion, this study result demonstrated that vitamin D supplementation was effective in reducing the RAGE expression. Furthermore, vitamin D could decrease AGEs and TNF-α serum levels in type 2 diabetic patients with vitamin D deficiency or insufficiency.

This study strengths include the homogeneity of population, the small number of missing, and the double blind randomized placebo-controlled design. In addition, this study limitation was short follow up period.

## Conclusion

This placebo-controlled double-blind randomized trial indicated that vitamin D supplementation could ameliorate AGEs–RAGE signaling pathway in T2DM patients. Vascular complications are more frequent in diabetes, and are associated with mortality and morbidity increased incidence in diabetic patients. Vitamin D supplementation has few side effects and may prevent from the vascular complications onset in diabetic patients by AGEs–RAGE interaction inhibiting.

## Data Availability

Not applicable.
